# DLL3 Immunohistochemical Expression in Neuroendocrine-Transformed *EGFR*-Mutant Lung Cancer and Two Cases of Tarlatamab Therapy

**DOI:** 10.1016/j.jtocrr.2025.100913

**Published:** 2025-09-30

**Authors:** Jacqueline V. Aredo, Surbhi Singhal, Gerald J. Berry, Farshad Moradi, Heather A. Wakelee, Nathaniel J. Myall, Kavitha J. Ramchandran, Millie Das, Carlos J. Suarez, Joel W. Neal, Jonathan W. Riess

**Affiliations:** a Department of Medicine, Division of Oncology, Stanford Cancer Institute, Stanford University School of Medicine, Stanford, California; b Department of Medicine, Division of Hematology/Oncology, University of California Davis Comprehensive Cancer Center, Sacramento, California; c Department of Pathology, Stanford University School of Medicine, Stanford, California; d Department of Radiology, Division of Nuclear Medicine, and Molecular Imaging, Stanford University School of Medicine, Stanford, California

**Keywords:** Neuroendocrine transformation, Small cell transformation, Tarlatamab, Osimertinib, EGFR mutation

## Abstract

**Introduction:**

Histologic transformation to high-grade neuroendocrine carcinoma occurs in resistance to EGFR targeted treatment in approximately 3% to 4% of patients with *EGFR*-mutant lung cancer and is associated with poor outcomes. The bispecific T-cell engager, tarlatamab, targets DLL3 and CD3 and has exhibited activity in classical SCLC. We evaluated DLL3 expression in patients with neuroendocrine-transformed *EGFR*-mutant lung cancer and present two cases who received tarlatamab.

**Methods:**

Patients with high-grade neuroendocrine *EGFR*-mutant lung cancer *de novo* or after treatment with osimertinib were evaluated at two academic centers. DLL3 expression in neuroendocrine tissue was assessed by immunohistochemistry using the VENTANA SP347 assay (Roche Diagnostics International AG, Rotkreuz, Switzerland).

**Results:**

Twelve patients were identified. Initial histologic diagnoses included adenocarcinoma (n = 10), adenosquamous (n = 1), and combined small cell carcinoma with an adenocarcinoma component (n = 1), with eight having *EGFR* exon 19 deletions and four with *EGFR* L858R. *TP53* co-mutations and *RB1* loss were detected in all patients tested (10 and 7, respectively). The median time from osimertinib initiation to neuroendocrine transformation was 27.8 months (range 3.6–52.9). DLL3 expression was positive in 11 patients with 15 samples (median 80%, range 1–100) and negative in one patient. Two patients with small cell transformation and 100% tumor DLL3 expression underwent treatment with tarlatamab with progression; osimertinib was subsequently added to tarlatamab in one patient with substantial improvement in all lesions.

**Conclusions:**

In this study, neuroendocrine-transformed *EGFR*-mutant lung cancer exhibited variable DLL3 expression. Tarlatamab appeared effective when added to osimertinib. Further analysis of the combination of bispecific DLL3 T-cell engager and EGFR tyrosine kinase inhibitor is warranted to confirm these findings.

## Introduction

Histologic transformation to high-grade neuroendocrine carcinoma—including small cell carcinoma—develops as a resistance mechanism to EGFR tyrosine kinase inhibitors (TKIs) in approximately 3% to 4% of patients with *EGFR*-mutant NSCLC.^1^^,^^2^ Neuroendocrine transformation is associated with poor outcomes,^3^ and is typically less responsive to immune checkpoint inhibitors combined with chemotherapy compared with classical SCLC.^4^^,^^5^ Chemotherapy, typically platinum-etoposide, is the backbone of treatment of neuroendocrine-transformed *EGFR*-mutant lung cancer. Although initial response rates are high at 46% to 54%, progression-free survival (PFS) is limited at 3 to 4 months, underscoring a need for improved therapies.^3^^,^^4^

Among the recent advances in the treatment of extensive-stage SCLC is the bispecific T-cell engager, tarlatamab, which targets DLL3 and CD3 to facilitate T-cell lysis of tumor cells.^6^ DLL3 acts by inhibiting the Notch signaling pathway and is up-regulated on the cell surface of SCLC tumor cells,^7^ making it an ideal therapeutic target. Tarlatamab has exhibited promising activity in patients with refractory SCLC,^8^ and is now a treatment option after platinum-based chemotherapy.^9^ Tarlatamab is also under investigation in other neuroendocrine neoplasms, such as neuroendocrine prostate cancer, in which DLL3-expressing tumors exhibited enhanced antitumor activity from tarlatamab compared with the cohort as a whole (objective responses in 22.2% versus 10.5%, respectively).^10^ Another phase II basket trial is investigating tarlatamab in DLL3-expressing tumors other than SCLC and neuroendocrine prostate cancer (NCT06788938). The effectiveness of tarlatamab in neuroendocrine-transformed *EGFR*-mutant lung cancer has not been established. We evaluated DLL3 expression in patients with neuroendocrine-transformed *EGFR*-mutant lung cancer and present two cases of tarlatamab treatment in *EGFR*-mutant SCLC.

## Materials and Methods

We identified patients diagnosed with *EGFR*-mutant NSCLC who received osimertinib and had biopsy-confirmed neuroendocrine histology *de novo* or as a resistance mechanism to therapy. Patients were identified by review of institutional databases in search of neuroendocrine transformation within *EGFR*-mutant NSCLC. Tissue histology was classified according to WHO guidelines.^11^ Neuroendocrine histology was confirmed morphologically and by positive immunohistochemical staining of neuroendocrine markers, including synaptophysin, chromogranin, and INSM1.

Molecular profiling was conducted using validated next-generation sequencing or targeted gene assays (Supplementary Table 1). Demographic, clinical, and pathologic data were abstracted from electronic health records. This study was performed at the Stanford Cancer Institute and UC Davis Comprehensive Cancer Center under respective institutional review board-approved protocols. Patients provided informed consent for study participation unless exempted, per the study protocols.

Overall survival (OS) was measured from the date of neuroendocrine transformation to death. Tumor response was evaluated radiographically using the Response Evaluation Criteria in Solid Tumors version 1.1.^12^ PFS was measured from treatment initiation to disease progression or death, whichever occurred first. Central nervous system PFS was measured from treatment initiation to central nervous system progression or death, whichever occurred first. The Kaplan-Meier method was used to estimate survival times and confidence intervals.

### DLL3 Immunohistochemistry

Formalin-fixed, paraffin-embedded slides of neuroendocrine-transformed tumor tissue were retrieved and stained with the DLL3 SP347 antibody using the VENTANA assay (Roche Diagnostics International AG, Rotkreuz, Switzerland) and viewed using the OptiView detection kit. DLL3 immunohistochemistry (IHC) staining intensity was classified as none (0), weak (1+), moderate (2+), and strong (3+). DLL3 staining was considered positive if any (≥1%) tumor cells exhibited cytoplasmic or membranous staining of DLL3 at any intensity, as previously described.^13^ The percentage of tumor cells staining positive for DLL3 was reported. As a negative control, DLL3 IHC staining was performed on the initial adenocarcinoma tissue samples of two patients with available tissue.

## Results

The cohort included 11 patients with neuroendocrine-transformed *EGFR*-mutant lung cancer and one patient with *de novo* *EGFR*-mutant SCLC. Patient characteristics are summarized in Supplementary Table 2. Most patients had an initial lung adenocarcinoma except one who had an adenosquamous carcinoma and one who had a combined small cell carcinoma with an adenocarcinoma component at diagnosis (Table 1); all tumors were TTF-1–positive. Eight patients had tumors harboring *EGFR* exon 19 deletions, and four had *EGFR* L858R mutations. *TP53* co-mutations were identified in 10 out of 10 tumors tested, and *RB1* loss was detected in seven out of seven tumors tested.

**Table 1:** DLL3 Immunohistochemistry by Tumor Histology and Molecular Profile

Patient ID	Baseline Histology	*EGFR* mutation	Co-alterations	Stage at Osimertinib Initiation	Osimertinib Line of Treatment	Time to NE Transformation (months)	NE Histology	Ki-67 (%)	DLL3 IHC Intensity	DLL3 IHC Positivity (%)
1	Adenocarcinoma	Exon 19 deletion	*TP53, RB1*^a^	IV	1	29.9	High-grade NEC	66	0	0
2^b^	Adenocarcinoma	Exon 19 deletion	*TP53*	IV	1	17.4	Small cell carcinoma	75	1+	1
							Small cell carcinoma	80	1+	5
3^b^	Adenocarcinoma	Exon 19 deletion	*TP53*	IV	1	48.8	High-grade NEC	75	1+	10
							Large cell NEC	60	1+	30
4	Adenosquamous carcinoma	Exon 19 deletion	*TP53, RB1*^a^	IV	1	22.9	Small cell carcinoma	86	2+	90
5	Adenocarcinoma	L858R	*TP53, RB1*^a^	IV	1	3.6	High-grade NEC	55	3+	80
6	Adenocarcinoma	Exon 19 deletion	Not evaluated	IV	1	30.3	High-grade NEC	85	3+	80
7	Adenocarcinoma	Exon 19 deletion, T790M	*TP53, EGFR* amp	IV	3	30.2	High-grade NEC	55	3+	95
8	Adenocarcinoma	L858R	*TP53, RB1*^a^*, SMARCA4, ATM, EGFR* amp	IV	1	27.8	High-grade NEC	70	3+	95
9^b^	Adenocarcinoma	Exon 19 deletion	Not evaluated	IV	1	52.9	Small cell carcinoma	70	2+	95
							Small cell carcinoma	50	3+	75
10	Adenocarcinoma	L858R, I744M	*TP53, RB1*^a^*, APC*	IIA	Adjuvant	6.8	Small cell carcinoma	NA	3+	100
11^b^	Adenocarcinoma	Exon 19 deletion	*TP53, RB1*^a^*, PIK3CA*	IV	1	13.1	Small cell carcinoma	80	2+	95
							Small cell carcinoma	85	3+	100
12	Combined Small Cell/Adenocarcinoma	L858R	*TP53, RB1*^a^*, EGFR* amp	IV	1	NA	Small cell Carcinoma	NA	3+	100

ID, identification; IHC, immunohistochemistry; NA, not available; NE, neuroendocrine; NEC, neuroendocrine carcinoma.

a *RB1* loss was detected in seven out of seven patients evaluated through next-generation sequencing or immunohistochemistry.

b Four patients had two biopsy samples of neuroendocrine-transformed tissue collected at separate time points; histologic and DLL3 data are presented for both samples.

Osimertinib was initiated for stage IV disease in 11 patients; 1 patient with stage IIA disease received adjuvant osimertinib (Table 1). The median time from osimertinib initiation to neuroendocrine transformation was 27.8 months (range 3.6–52.9), and the median OS from neuroendocrine transformation was 15.6 months (range 2.2–32.9) (Supplementary Fig. 1*A* and *B*). Additional treatment outcomes are summarized in Supplementary Figure 1*C* to *E*.

**Figure 1:**
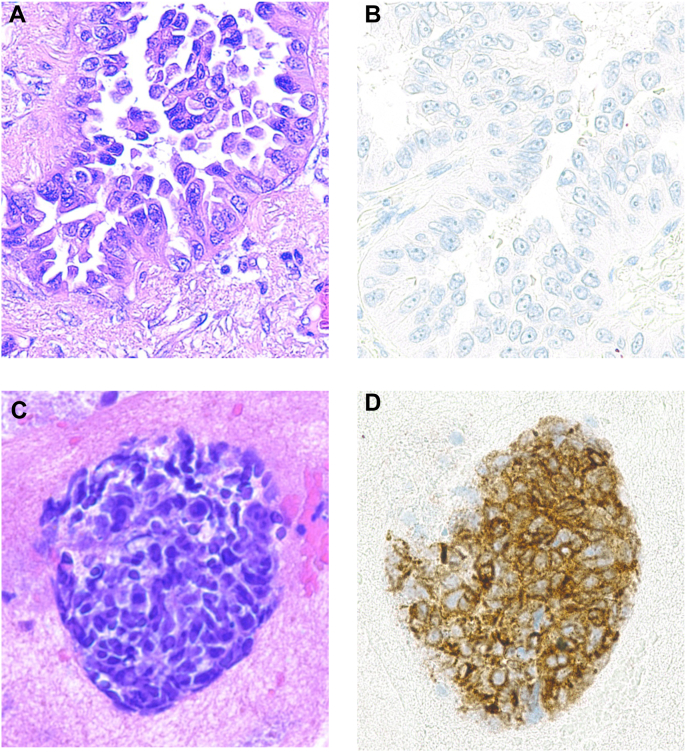
DLL3 Staining of *EGFR*-Mutant Lung Adenocarcinoma and Neuroendocrine-Transformed Carcinoma. Histologic images of a patient’s baseline *EGFR*-mutant lung adenocarcinoma (top row, *A-B*) and neuroendocrine-transformed carcinoma (bottom row, *C-D*). Hematoxylin and eosin staining was performed on the images in the left column (*A, C*), and DLL3 staining was performed on the images in the right column (*B, D*). In this patient, the pulmonary adenocarcinoma tissue (*B*) stained with zero intensity in both the membrane and cytoplasm, with 0% of cells staining positive for DLL3. The left parietal brain exhibiting high-grade neuroendocrine-transformed tissue (*D*) stained with 3+ intensity in both the membrane and cytoplasm, with 100% of tumor cells staining positive for DLL3. All magnifications are at x200.

Histologic transformation was evaluated in 16 tissue samples, including four patients with two biopsy samples collected at separate time points (Table 1, Fig. 1, Supplementary Table 2). Nine samples revealed small cell carcinoma, six exhibited high-grade neuroendocrine carcinoma not otherwise specified, and one exhibited large cell neuroendocrine carcinoma. Nine tumors underwent repeat molecular sequencing posttransformation, and all retained the founder *EGFR* and *TP53* mutations. DLL3 IHC was positive in 11 out of 12 (91.7%) patients, and the median DLL3 positivity was 80% (range 0–100). Eight patients had at least one tissue sample staining DLL3 at 3+ intensity. In contrast, the two initial adenocarcinoma samples stained negative for DLL3 (0 intensity, 0% expression).

### Case 1

A 51-year-old never-smoking woman presented with progressive cough. Imaging revealed a left lower lobe lung mass, a pancreatic mass, and hilar and mediastinal lymphadenopathy that were all FDG-avid on positron emission tomography with computed tomography scan (PET/CT). Biopsy of the lung mass revealed combined small cell carcinoma with an adenocarcinoma component (TTF-1+/CK7+). Plasma circulating tumor DNA revealed an *EGFR* L858R mutation, *TP53* mutation, *EGFR* amplification, and *RB1* loss. The patient was initiated on carboplatin-etoposide and osimertinib with progressive disease after approximately 3 months with new brain metastases treated with stereotactic radiation and new liver lesions (Supplementary Fig. 2). DLL3 IHC was performed on the baseline biopsy, and 100% expression was noted. The patient was then initiated on tarlatamab with further progressive disease after approximately 2 months. She then elected to proceed with comfort care.

### Case 2

**Figure 2:**
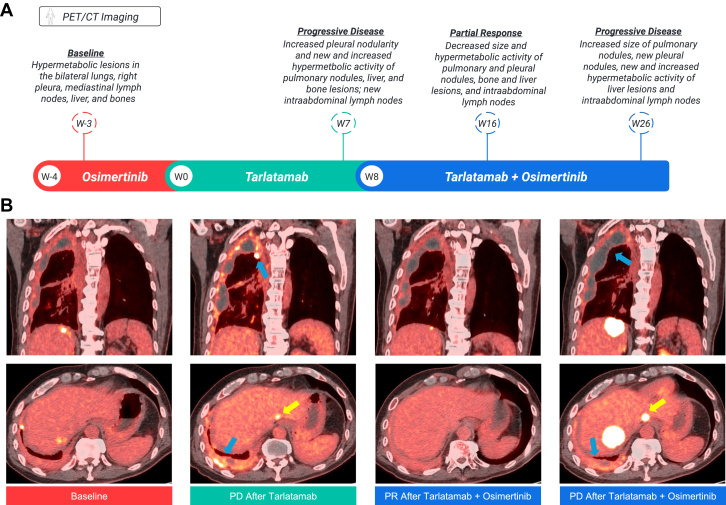
Case 2 Timeline and Imaging. (*A*) Patient treatment and PET/CT imaging timeline. (*B*) Sequential PET/CT scans from: baseline (left column) when the patient was on bridging osimertinib with the scans notable for right pleural nodularity and scattered hepatic metastases; second scans (second column) after tarlatamab revealing progressive disease with increased right pleural hypermetabolic activity and nodularity (blue arrows) and a new hepatic lesion (yellow arrow); third scans (third column) after osimertinib was added to tarlatamab exhibiting a partial response to all lesions; and final scans (fourth column) with tarlatamab and osimertinib illustrating disease progression with increased pleural nodularity (blue arrows) and growing (yellow arrow) and new hepatic lesions. PET/CT, positron emission tomography/computed tomography; W week; PD progressive disease; PR partial response.

A 65-year-old man presented with cough and dyspnea and was diagnosed with stage IV *EGFR* exon 19 deletion well-differentiated lung adenocarcinoma (TTF-1+/CK7+), with metastases involving the right pleura, bilateral lungs, mediastinal lymph nodes, and bones (Fig. 2). He initiated first-line osimertinib with a partial response. Disease progression occurred after 12.5 months, and repeat lung biopsy revealed small cell carcinoma (TTF-1+/synaptophysin+/INSM1+; Ki-67 85%). The patient stopped osimertinib and initiated carboplatin-etoposide followed by consolidative radiation. When progression occurred, he received cisplatin-irinotecan with progression after 5.5 months involving new liver and brain metastases. He temporarily resumed osimertinib as bridging therapy while DLL3 IHC testing was performed.

His tumor DLL3 IHC returned positive with 3+ staining and 100% expression. He then initiated tarlatamab at standard dosing. Each dose was complicated by grade 1 cytokine release syndrome. PET/CT after two cycles revealed disease progression with new liver and bone lesions, increased right pleural nodularity, and new intraabdominal lymphadenopathy. Osimertinib was added to tarlatamab and repeat PET/CT 8 weeks later revealed substantial tumor shrinkage of the previously growing pulmonary and pleural nodules, and the hepatic, intraabdominal, and osseous lesions. Progression occurred after a total of 6 months of tarlatamab treatment. Repeat biopsy of a progressive liver metastasis redemonstrated the small cell carcinoma without a clear adenocarcinoma component.

## Discussion

In this study, we identified variable expression of DLL3 by IHC in neuroendocrine-transformed tissue of *EGFR*-mutant lung cancer resistant to osimertinib. Yet, 8 out of 12 (67%) patients had samples exhibiting strong (3+) DLL3 staining, and 9 out of 12 (75%) patients had DLL3 expression of at least 75%, supporting investigation of DLL3-directed therapies in this treatment-resistant setting in which few effective therapies exist. The cohort characteristics were representative of those previously described with neuroendocrine-transformed *EGFR*-mutant lung cancer.^3^^,^^5^ One previous analysis of DLL3 expression in SCLC transformed tissue found DLL3 expression in 93% of specimens, which was absent in baseline adenocarcinoma samples.^4^

The tarlatamab cases presented were *de novo* and acquired histologic transformation of SCLC, both with 100% tumor DLL3 expression. Tarlatamab monotherapy led to rapid progression in both patients, but then a substantial response occurred in the patient in whom osimertinib was added to tarlatamab. There are two potential explanations for this combination effect. First, resensitization to EGFR TKI from existing adenocarcinoma clones that persist after histologic transformation. This hypothesis is supported by a previous study in which biopsy confirmed adenocarcinoma in progressive lesions after small cell transformation in a subset of patients.^3^ In case 2, repeat biopsy after progression on osimertinib and tarlatamab revealed only residual SCLC. In addition to its activity on the *EGFR*-mutant receptor in adenocarcinoma, EGFR TKIs have been found to modulate the immune microenvironment and enhance cytotoxic T-cell killing.^14^^,^^15^ After osimertinib was resumed in this case, there was a widespread response to therapy, disproportionate to what would be expected with a small number of adenocarcinoma clones in transformed SCLC. Therefore, it is possible that favorable immune cell alterations and enhanced cytotoxic T-cell activity of the EGFR TKI potentiates the tarlatamab activity against small cell clones. The total duration of tarlatamab treatment in this patient was 6 months. Tarlatamab was safely combined with osimertinib and well-tolerated with no unexpected adverse events.

This study is limited by its retrospective nature and small sample size. Nevertheless, the presence of DLL3 expression in neuroendocrine-transformed tissue and the case studies support further investigation of a DLL3-targeting agent and EGFR TKI in neuroendocrine-transformed *EGFR*-mutant lung cancer. Future initiatives should aim to characterize molecular markers of response and immune cell alterations to combination DLL3 bispecific T-cell engagers and EGFR TKIs to guide improved therapy for this challenging disease.

## CRediT Authorship Contribution Statement

**Jacqueline V. Aredo:** Conceptualization, Methodology, Investigation, Data Curation, Formal Analysis, Visualization, Writing – Original Draft, Writing – Review & Editing.

**Surbhi Singhal:** Conceptualization, Investigation, Writing – Review & Editing, Resources.

**Gerald J. Berry:** Investigation, Visualization, Writing – Review & Editing, Resources.

**Farshad Moradi:** Investigation, Visualization, Writing – Review & Editing, Resources.

**Heather A. Wakelee:** Writing – Review & Editing, Resources.

**Nathaniel J. Myall:** Writing – Review & Editing, Resources.

**Kavitha J. Ramchandran:** Writing – Review & Editing, Resources.

**Millie Das:** Writing – Review & Editing, Resources

**Carlos Jose Suarez:** Writing – Review & Editing, Resources.

**Joel W. Neal:** Conceptualization, Methodology, Writing – Original Draft, Writing – Review & Editing, Resources, Supervision.

**Jonathan W. Riess:** Conceptualization, Methodology, Investigation, Visualization, Writing – Original Draft, Writing – Review & Editing, Resources, Supervision.

## Disclosure

Dr. Aredo reports receiving consulting fees from AstraZeneca; has served on the advisory boards of Johnson & Johnson and Bristol Myers Squibb; and reports receiving honoraria from Kaplan, Binaytara Foundation, and IDEOlogy Health. Dr. Singhal reports receiving research support from the National Institutes of Health and consulting fees from OncoHost; and has served on the advisory boards of Caris Life Sciences and Bristol Myers Squibb. Dr. Wakelee reports receiving research support to the institution from AstraZeneca, Bayer, Bristol Myers Squibb, Genentech/Roche, Helsinn, Merck, SeaGen, Xcovery, and Gilead by means of investigator-initiated trial (Georgetown); consulting fees from IOBiotech, Mirati, OncoC4, Beigene (BeOne), and GSK; had unpaid advisory roles from Bristol Myers Squibb, Genentech/Roche, Merck, and AstraZeneca; and received travel support from Chugai. Dr. Myall reports receiving research support from Genentech. Dr. Ramchandran reports receiving research support from Varian. Dr. Das reports receiving research support from Merck, Genentech, CellSight, Novartis, and Varian; consulting fees from Eurofins, Abbvie, Janssen, Gilead, Daiichi Sankyo, and Bristol Myers Squibb; and has served on the advisory boards of Sanofi/Genzyme, Regeneron, Janssen, AstraZeneca, Gilead, Bristol Myers Squibb, Catalyst Pharmaceuticals, Novocure, Guardant, EMD Sereno, Natera, Merus, OncoHost, Jazz Pharmaceuticals, Summit, Novartis, and Jazz. Dr. Neal reports receiving research support from Revolution Medicines, Nuvalent, Inc., Genentech/Roche, Merck, Novartis, Boehringer Ingelheim, Exelixis, Nektar Therapeutics, Takeda Pharmaceuticals, Adaptimmune, GSK, Janssen, and Gilead; consulting fees from AstraZeneca, Genentech/Roche, Exelixis, Takeda Pharmaceuticals, Eli Lilly and Company, Amgen, Iovance Biotherapeutics, Blueprint Pharmaceuticals, Regeneron Pharmaceuticals, Natera, Sanofi/Regeneron, D2G Oncology, Surface Oncology, Turning Point Therapeutics, Mirati Therapeutics, Gilead Sciences, AbbVie, Summit Therapeutics, Novartis, Novocure, Janssen Oncology, Anheart Therapeutics, Bristol Myers Squibb, Daiichi Sankyo/AstraZeneca, and Nuvation Bio; and honoraria from CME Matters, Clinical Care Options CME, Research to Practice CME, Medscape CME, Biomedical Learning Institute CME, MLI Peerview CME, Prime Oncology CME, Projects in Knowledge CME, Rockpointe CME, MJH Life Sciences CME, and Medical Educator Consortium. Dr. Riess reports receiving research support from Merck, Novartis Pharmaceuticals, Revolution Medicine, AstraZeneca, Arrivent, IO Biotech, Summit, Nuvalent, SeaGen, Prelude, and Blossom Hill; consulting fees from OncoHost; and has served on the advisory boards of AstraZeneca, Boehringer Ingelheim, Bristol Myers Squibb, Daiichi Sankyo, GSK, Janssen Pharmaceuticals, Merck, Bicycle Therapeutics, Nuvation, and Pfizer.

## Supplementary Data

Supplementary_Table_1

Supplmentary_Table_2
