# Supplementary material for: DLL3 Immunohistochemical Expression in Neuroendocrine-Transformed EGFR-Mutant Lung Cancer and Two Cases of Tarlatamab Therapy
Source: JTO Clin Res Rep. 2025 Sep 30;6(12):100913. doi: 10.1016/j.jtocrr.2025.100913 (PMC12621428; doi:10.1016/j.jtocrr.2025.100913)

Supplementary Figure 1. Cohort Outcomes.
(A) Cumulative incidence of time to neuroendocrine transformation, (B) overall survival from neuroendocrine transformation, and (C) progression-free survival and (D) central nervous system progression-free survival from first therapy. (E) Progression-free survival did not significantly differ by level of DLL3 expression. No/low DLL3 expression was defined by <50% of tumor cells staining positive for DLL3 on immunohistochemistry of neuroendocrine transformed tissue, whereas high DLL3 expression was defined by ≥50% of tumor cells staining positive for DLL3.
Abbreviations: *NE* neuroendocrine, *No* number, *CI* confidence interval, *OS* overall survival, *NE* not estimable, *PFS* progression-free survival, *DLL3* delta-like ligand 3.

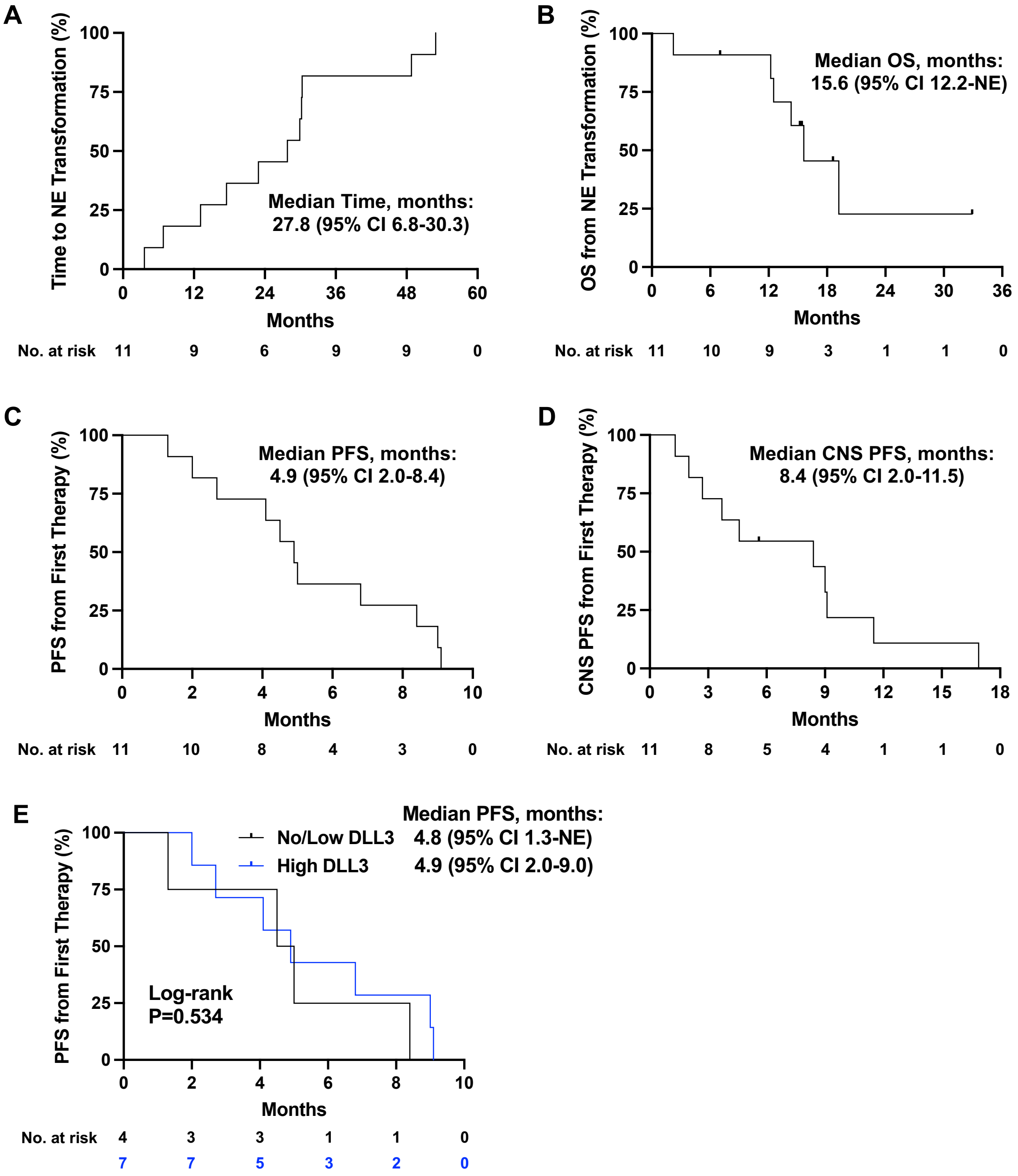

Supplement: Supplementary_Figure_1 [file mmc1.pdf]
