# Supplementary material for: DLL3 Immunohistochemical Expression in Neuroendocrine-Transformed EGFR-Mutant Lung Cancer and Two Cases of Tarlatamab Therapy
Source: JTO Clin Res Rep. 2025 Sep 30;6(12):100913. doi: 10.1016/j.jtocrr.2025.100913 (PMC12621428; doi:10.1016/j.jtocrr.2025.100913)

Supplementary Figure 2. Case 1 Imaging.
Sequential CT scans of the liver from: baseline (first panel) without any visualizable hepatic metastases; second scan (second panel) after carboplatin/etoposide and osimertinib showing progression with new hepatic metastases; and final scan (third panel) after tarlatamab showing ongoing progression with new and increased size of the hepatic metastases.
Abbreviation: *PD* progressive disease.

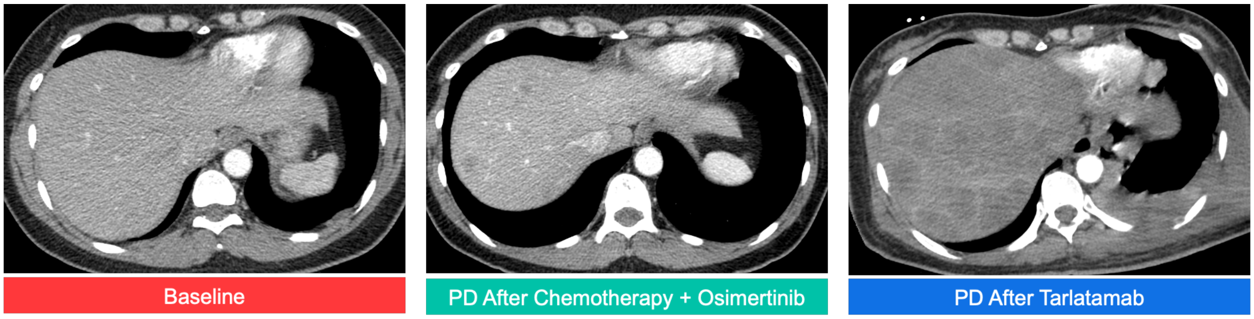

Supplement: Supplementary_Figure_2 [file mmc2.pdf]
