# Supplementary material for: DLL3 Immunohistochemical Expression in Neuroendocrine-Transformed EGFR-Mutant Lung Cancer and Two Cases of Tarlatamab Therapy
Source: JTO Clin Res Rep. 2025 Sep 30;6(12):100913. doi: 10.1016/j.jtocrr.2025.100913 (PMC12621428; doi:10.1016/j.jtocrr.2025.100913)
Supplement: Supplementary_Table_1 [file mmc3.pdf]

Supplementary Table 1. List of Molecular Platforms for Baseline Tumor Sequencing.

**Platform**	**N=12**
Stanford Actionable Mutation Panel for Solid Tumors	5
FoundationOne CDx	4
Guardant360 Liquid	1
Lung Cancer Mutation Panel	1
EGFR Polymerase Chain Reaction	1
