# Supplementary material for: DLL3 Immunohistochemical Expression in Neuroendocrine-Transformed EGFR-Mutant Lung Cancer and Two Cases of Tarlatamab Therapy
Source: JTO Clin Res Rep. 2025 Sep 30;6(12):100913. doi: 10.1016/j.jtocrr.2025.100913 (PMC12621428; doi:10.1016/j.jtocrr.2025.100913)
Supplement: Supplmentary_Table_2 [file mmc4.pdf]

Supplementary Table 2. Patient Characteristics

Characteristic	N=12
Age, median (range), years	57.0 (36-66)
Sex, N (%)	
Female	7 (58.3)
Male	5 (41.7)
Race/ethnicity, N (%)	
Asian	7 (58.3)
White	4 (33.3)
Hispanic	1 (8.3)
Smoking Status, N (%)	
Never	9 (75.0)
Former	3 (25.0)
Initial Tumor Histology, N (%)	
Adenocarcinoma	10 (83.3)
Adenosquamous carcinoma	1 (8.3)
Combined small cell carcinoma with an adenocarcinoma component	1 (8.3)
Initial Tumor Stage, N (%)	
IIA	1 (8.3)
IV	11 (91.7)
Brain Metastases at Diagnosis, N (%)	
No	8 (66.7)
Yes	4 (33.3)
Osimertinib Line of Treatment, N (%)	
1	10 (83.3)
3	1 (8.3)
Adjuvant	1 (8.3)
Time to Neuroendocrine Transformation, median (range), months	27.8 (3.6-52.9)
Neuroendocrine Histology, N (%)[a]	
Small cell carcinoma	9 (56.3)
High-grade neuroendocrine carcinoma NOS	6 (37.5)
Large cell neuroendocrine carcinoma	1 (6.3)
Ki-67, median (range), %[a]	70 (50-86)
DLL3 IHC Intensity, N (%)[a]	
0	1 (6.3)
1+	4 (25.0)
2+	3 (18.8)
3+	8 (50.0)
DLL3 IHC Positivity, median (range), %[a]	80 (0-100)
First Therapy for Neuroendocrine Disease, N (%)	
Carboplatin/etoposide	6 (50.0)
Carboplatin/etoposide/atezolizumab	2 (16.7)
Concurrent chemoradiotherapy with platinum/etoposide	2 (16.7)
Carboplatin/paclitaxel/bevacizumab	1 (8.3)
Temozolomide/olaparib	1 (8.3)
Osimertinib Continued for Neuroendocrine Disease, N (%)	
No	6 (50.0)
Yes	6 (50.0)

Abbreviations: *N* sample size, *DLL3* delta-like ligand 3, *NOS* not otherwise specified, *IHC* immunohistochemistry.

[a] Neuroendocrine histology, Ki-67, and DLL3 IHC were assessed in N=16 tissue samples with available data, including 4 patients who had 2 neuroendocrine tissue samples collected at separate timepoints.
